# Glutathione provides antioxidative defence and promotes microspore-derived embryo development in isolated microspore cultures of triticale (× *Triticosecale* Wittm.)

**DOI:** 10.1007/s00299-018-2362-x

**Published:** 2018-11-29

**Authors:** Iwona Żur, Ewa Dubas, Monika Krzewska, Kamil Zieliński, Jozsef Fodor, Franciszek Janowiak

**Affiliations:** 1 The Franciszek Górski Institute of Plant Physiology, Polish Academy of Sciences, Niezapominajek 21, 30-239 Kraków, Poland; 2 Plant Protection Institute, Hungarian Academy of Sciences, Herman Ottó út 15, Budapest, 1022 Hungary

**Keywords:** Antioxidative activity, Glutathione, Microspore embryogenesis, Oxidative stress, Triticale

## Abstract

**Key message:**

Depending on the capability for stress adaptation, the role played by glutathione in microspore embryogenesis consists of both antioxidative activity and stimulation of embryo-like structure development.

**Abstract:**

The efficiency of microspore embryogenesis (ME) is determined by the complex network of internal and environmental factors. Among them, the efficient defence against oxidative stress seems to be one of the most important. The present study confirms this hypothesis showing the positive effect of glutathione—the most abundant cellular antioxidant—on ME in isolated microspore cultures of triticale (× *Triticosecale* Wittm.). For the first time, low temperature (LT) pre-treatment of tillers was combined with the exogenous application of glutathione and associated with the total activity of low-molecular weight antioxidants, the endogenous content and redox status of glutathione, and the effectiveness of ME. The results indicate that efficient antioxidative defence is the first, although not the only, prerequisite for effective ME. In responsive genotypes, LT alone stimulated antioxidative defence and decreased cell redox status, which was associated with increased cell viability and high frequency (ca. 20%) of microspore reprogramming. Application of glutathione had no effect either on the microspore viability or on the initial number of embryogenic microspores. However, it increased the number of embryo-like structures, probably by stimulating the next phases of its development. In recalcitrant genotypes, the main role of glutathione seems to be its participation in cell protection from oxidative stress. However, even enhanced antioxidative activity, which sustained cell viability and increased the number of embryogenic microspores, was insufficient for efficient haploid/doubled haploid plant production. Evidently, there are still other defective elements in the complex network of factors that regulate the process of ME.

**Electronic supplementary material:**

The online version of this article (10.1007/s00299-018-2362-x) contains supplementary material, which is available to authorized users.

## Introduction

The process of microspore embryogenesis (ME) is an alternative pathway of development, available for in vitro cultured immature pollen grains (microspores), which leads to the formation of haploid embryo-like structures (ELSs), able to regenerate haploids/doubled haploids (DHs). As the shortest way to total homozygosity, it attracts a lot of attention, especially due to the benefits of its utilisation in plant breeding (Kasha and Maluszynski 2003). However, in the case of many plant species, including triticale (× *Triticosecale* Wittm.), highly differentiated genotype-dependent effectiveness of the process limits its exploitation on a large, commercial scale. Despite extensive examination, the precise mechanism of ME induction and factors important for effective DH formation are not yet fully recognized. The reason for this setback is the complexity and multifactorial nature of the process, which depends significantly on both endogenous and environmental factors and on an intricate network of their specific interactions.

Due to the fact that ME is induced or at least strongly stimulated by a stress pre-treatment (Touraev et al. 1997a, b; Zoriniants et al. 2005; Shariatpanahi et al. 2006), high stress tolerance could be presumed the first prerequisite for successful ME induction. One of the most important components of stress tolerance is an effective antioxidative system composed of both enzymatic and low-molecular weight (LMW) antioxidants, protecting the cells from the effects of reactive oxygen species (ROS) generation (Mittler 2002). Our earlier investigations revealed that the activity of antioxidative enzymes measured in triticale anthers was genotype-dependent and significantly associated with the embryogenic potential, whereas the role of LMW antioxidants seemed to be less important (Żur et al. 2014a). However, several published reports showed positive effects of exogenously applied LMW antioxidants on ME effectiveness (Stasolla et al. 2008; Cistué et al. 2009; Asif et al. 2013; Zeng et al. 2017). In parallel, in several plant species gene-encoding enzymes involved in antioxidative defence [glutathione-*S*-transferase (GST), oxalate oxidase] were up-regulated in response to the treatment initiating ME (Vrinten et al. 1999; Jacquard et al. 2009; Sánchez-Díaz et al. 2013; Żur et al. 2014b). Increased accumulation of proteins typical for cell defence against oxidative stress (e.g., GST, l-ascorbate peroxidase) during ME induction was also reported (Cordewener et al. 2009; Uváčková et al. 2012; Krzewska et al. 2017).

Among LMW antioxidants, glutathione is the most abundant, found in most prokaryotes and eukaryotes. Within cells, it occurs predominantly in its reduced form (GSH) considered to be one of the most important ROS scavengers. In redox reactions, GSH is continuously oxidized into glutathione disulphide (GSSG), which can be rapidly converted back to its reduced form by glutathione reductase. The ratio between GSH and GSSG, the so-called ‘glutathione redox status’, has a big impact on cell redox balance and could be used as a parameter of the intensity of oxidative stress. Beyond this, glutathione plays a role in many other physiological processes, e.g., in biosynthesis, detoxification and antioxidant biochemistry (Noctor et al. 2012). It also acts as a signalling molecule involved in the regulation of cell cycle, proliferation and programmed cell death.

In the present study, the effect of GSH on the effectiveness of ME initiation was studied in isolated microspore cultures of several DH lines of triticale (× *Triticosecale* Wittm.) significantly different in respect of their embryogenic potential. For the first time, GSH pre-treatment was applied to tillers and combined with low temperature (LT) pre-treatment standardly used for ME initiation in triticale. The changes in the total activity of LMW antioxidants and the endogenous pool of glutathione together with the ratio between its reduced and oxidized forms were estimated and associated with the effectiveness of ME induction.

## Materials and methods

DH lines of winter triticale (× *Triticosecale* Wittm.) used in the study originated from a cross between the German breeding line ‘Saka3006’ (SaKa Pflanzenzucht GbR, Germany) and the Polish cultivar ‘Modus’ (Plant Breeding Strzelce Ltd.). The ‘Saka3006’ × ‘Modus’ mapping population composed of 90 DH lines was produced at Hohenheim University (Stuttgart, Germany) and kindly provided by Dr Eva Bauer. Seven DH lines studied in the first experiment were recognized earlier with the use of anther culture method as highly differentiated in respect of their embryogenic potential (Żur et al. 2014a, b, 2015; Krzewska et al. 2015). Five of them (DH18, DH19, DH28, DH47 and DH119) were used in a more detailed study as the object of biochemical analyses.

### Plant growth conditions

Germinating kernels were placed in perlite pre-soaked with Hoagland solution (HS) and vernalized for 7 weeks at 4 °C and 8 h/16 h (day/night) photoperiod. Vernalized seedlings were planted into pots containing a mixture of soil, de-acidified substrate peat and sand (2/2/1; v/v/v), and grown in a greenhouse at 25 °C and 16 h/8 h photoperiod.

### Pre-treatment of tillers used for microspore embryogenesis induction

Tillers from donor plants were collected when the majority of microspores were at mid- to late uni-nucleate stage of development, placed in HS medium and stored for 3 weeks at 4 °C in the dark. Simultaneously with low temperature pre-treatment, a part of tillers was also treated with 0.3 mM reduced glutathione (GSH) supplemented to HS medium. The concentration of GSH used in the study was chosen based on the results of a preliminary experiment in which concentrations in the range between 0.3 and 1 mM were tested (data not shown).

In the first experiment, cut tillers were exposed to: (1) low temperature (4 °C) for 21 days (LT), (2) LT and GSH, with GSH supplemented to HS medium at the start of LT pre-treatment (LT + GSH) or (3) LT and GSH, with GSH supplemented to HS medium on the last 3–8 days before microspore isolation (LT + 3–8d GSH).

In the second experiment, the above-mentioned tiller treatments with LT and LT + GSH were again applied to five DH lines of triticale. Additionally, the combination of LT and modified, short GSH pre-treatment, supplemented to HS medium precisely on the last 4 days before microspore isolation (LT + 4dGSH) was used for two DH lines of triticale (DH19, DH47).

The scheme of the experiment is presented in Online Resource 1.

### Microspore isolation and culture

The spikes were sprayed with 96% ethanol, surface sterilized in a 20% solution of commercial bleach (‘Domestos’) for 15 min and rinsed 4–5 times with sterile deionised water. Then, the spikes were cut into ca. 2 cm segments and blended in 0.3 M mannitol with the use of Waring blender (Fisher Scientific Inc.). The resulting slurry was filtered through a 74 µm metal sieve (200 mesh; CD-1, Sigma-Aldrich) and pelleted (100×*g*, 5 min). After removing the supernatant, the microspores were resuspended in 0.3 M mannitol and gently layered onto a 21% maltose solution for density gradient centrifugation (80×*g*, 5 min). Viable microspores settled at the interface between mannitol and maltose were collected, washed in 0.3 M mannitol and pelleted again (100×*g*, 5 min). Then, the supernatant was removed and the pelleted microspores were resuspended in 1 ml modified 190-2 medium (Zhuang and Xu 1983, modified according to; Pauk et al. 2000). The total number of collected microspores was estimated with a Neubauer counting chamber and then the suspension was sampled for in vitro culture and biochemical analyses. Microspore suspension with the final density of approximately 70 × 10^3^ microspores per ml (mcs/ml) was transferred to 15 × 60 mm petri dishes and co-cultured with immature ovaries (10 per 1 ml of suspension) which have been dissected simultaneously with microspore isolation. The cultures were incubated in the dark at 26 °C.

Starting from the sixth week of culture, embryo-like structures (ELS) of 1 mm in size were transferred onto 0.6% agar solidified regeneration medium 190-2 (Zhuang and Xu 1983) supplemented with 0.5 mg/l kinetin, 0.5 mg/l NAA and 30 g/l sucrose, pH 6.0. The cultures were kept at 26 °C, with 16 h/8 h (day/night) photoperiod in a dim light [80–100 µmol (hν) m^−2^ s^−1^ (PAR)].

### The measurements of isolated microspore yield, viability, and the frequency of embryogenesis initiation as well as the visualization of further stages of embryogenic development

Usually 5–6 spikes were used for one isolation procedure. The number of isolated microspores was estimated with the use of a Neubauer counting chamber. The number of isolated microspores received per one spike of a donor plant was considered as ‘isolated microspore yield’ (*Y*).

Microspore viability (*V*) was determined on the isolation day by fluorochromatic reaction to fluorescein diacetate (FDA; 0.01%; *λ*_Ex_ = 465 nm, *λ*_Em_ = 515 nm, green fluorescence; Heslop-Harrison and Heslop-Harrison 1970). The samples were examined under Nikon Eclipse-E600 equipped with a differential interference contrast (DIC) system. Images were collected with Nikon DS-Ri1 digital camera and processed with NIS-Elements AR 3.0 Imaging Analysis, Microsoft PowerPoint and Corel PhotoPaint 10.0.

The frequency of embryogenic microspores was assessed on the isolation day with an inverted light microscope (NIKON TS-100/100F) by calculating the percentage of star-like structures (SLS) and microspores undergoing the first symmetrical division, as typical hallmarks of microspore embryogenesis initiation (Touraev et al. 1997a; Indrianto et al. 2001; Dubas et al. 2010). Next phases of embryogenic development were examined microscopically in petri dishes containing microspore suspensions after 7, 14 and 21 days of in vitro culture.

### Total antioxidant activity estimated by DPPH method

Isolated microspores (samples of ca. 70 mg fresh weight) were immediately frozen in liquid nitrogen and then stored until the analyses in a deep freezer at − 60 °C. The samples were freeze dried and ground with ball mill MM400 (Retsch, Haan, Germany) in Eppendorf vials, to which 1 ml of 50% ethanol was then added, and shaken for two hours at room temperature. The extracts were centrifuged for 20 min in a centrifuge at 18,000×*g* (MPW-350R, Warsaw, Poland) and the supernatant was used for the measurements. Total antioxidant activity (free radical-scavenging activity) in the tissues was measured by DPPH method according to Brand-Williams et al. (1995) with some modifications adapting the protocol to 96-well microtitre plates and to the measurement of absorbance with a microtitre plate reader (Płażek et al. 2011). A solution of 0.5 mM of stable free radical 1,1-diphenyl-2-picrylhydrazyl (DPPH, SIGMA) in methanol was used. Absorbance was determined after 30 min of the reaction at 37 °C at 515 nm using reader Model 680 (Bio-Rad Laboratories, Hercules, CA, USA). The results are expressed as µmoles of Trolox (SIGMA) equivalents. For each pre-treatment, at least three independent measurements were made.

### Measurement of reduced (GSH) and oxidized (GSSG) glutathione

The collected microspores were homogenized in ice-cold 6% meta-phosphoric acid and centrifuged for 20 min at 12,000×*g*, at 4 °C.

Total glutathione was determined by the method of Knörzer et al. (1996). Samples (100 µl supernatant) were neutralized with 25 µl 50 mM potassium phosphate buffer (pH 7.5) and 25 µl triethanolamine. The assay mix contained 20 µl neutralized plant extract and up to 900 µl potassium phosphate buffer (pH 7.5) containing 1 mM dithio-bis-nitrobenzoic acid, 0.2 mM NADPH, and 1 unit glutathione reductase (GR). The absorbance was recorded at 412 nm for 3 min The activity of GR enzyme is proportional with glutathione concentrations. Control reaction was carried out without plant extract. Glutathione concentration was calculated from the calibration curve prepared with GSH solutions of known concentrations.

Oxidized glutathione (GSSG) was determined by the same method (Knörzer et al. 1996) with the exception that reduced glutathione (GSH) was blocked with 2-vinylpyridine in the supernatant. 8 µl of 2-vinylpyridine were added to the neutralized extract, mixed and incubated for 1 h at room temperature. Then, 50 µl of neutralized supernatant was added to the assay mixture. Reduced GSH was calculated from the difference between total and GSSG concentrations. For each pre-treatment, at least three independent measurements were made.

The biochemical analyses were conducted on microspores isolated from (1) LT pre-treated tillers after 21 days at 4 °C in HS and (2) LT + GSH pre-treated tillers after 21 days at 4 °C in HS supplemented with 0.3 mM GSH. For two DH lines (DH19 and DH47), the effect of 4-day GSH pre-treatment (LT + 4dGSH) was also analysed. Additionally, freshly cut, non-treated tillers (NT) were used as control.

### Statistical analysis

All data after testing for normal distribution were examined by two-way analysis of variance (ANOVA), after which post hoc comparison was conducted using Duncan’s multiple range test (*p* ≤ 0.05). Nonparametric Spearman’s rank-order correlation coefficients (*R*) were used to visualize interrelationships among the studied traits. All statistical analyses were performed using STATISTICA version 12 (Stat Soft Inc.) package.

## Results

### Experiment 1

**Fig. 1:**
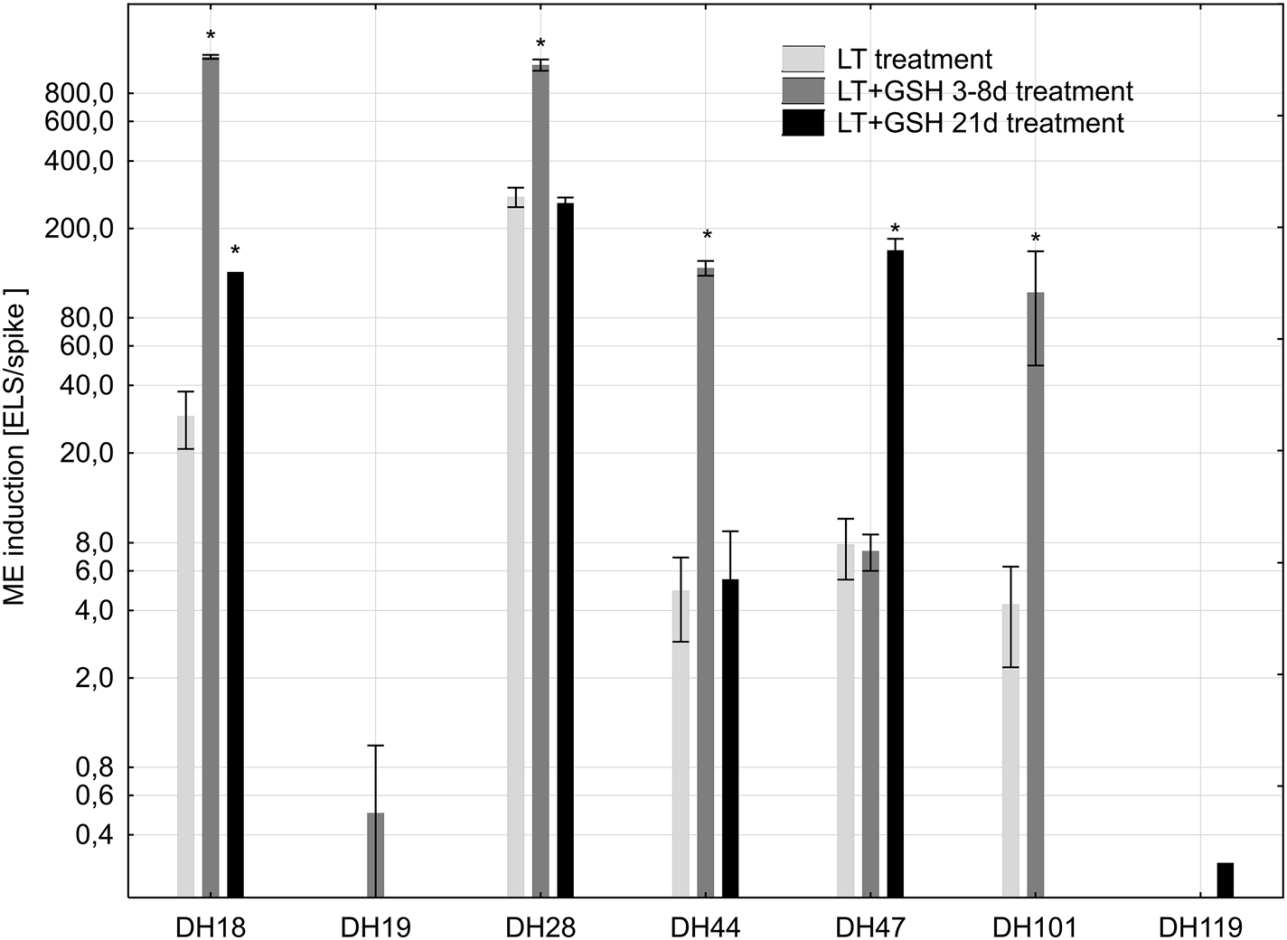
The effectiveness of microspore embryogenesis (mean number of embryo-like structures produced per spike of the donor plant ± SE) in isolated microspore cultures of winter triticale as the effect of various pre-treatments of tillers. The mean from at least three biological replications (isolations) ± SE. Axis *Y* is in the log scale. The asterisk symbol ‘*’ indicates values that are statistically different (*p* ≤ 0.05) from control (LT pre-treatment of tillers). *LT* low temperature pre-treatment of tillers (21 days at 4 °C); *LT + 3–8d GSH* low temperature pre-treatment of tillers (21 days at 4 °C) combined with short GSH pre-treatment (0.3 mM GSH applied 3–8 days before microspore isolation); *LT + GSH* low temperature pre-treatment of tillers (21 days at 4 °C) combined with long GSH pre-treatment (0.3 mM GSH applied 21 days before microspore isolation)

Low temperature pre-treatment of tillers turned out to be critical for successful isolation and transfer of triticale microspores to in vitro conditions. Microspores isolated from freshly cut, NT tillers usually did not survive the isolation procedure or died during the first days of in vitro culture. The GSH pre-treatment had no effect on the mean yield of isolated microspores, but significantly increased the efficiency of ME (Fig. 1). Depending on the genotype, short (3–8 days) and/or long (21 days) GSH pre-treatments of tillers markedly stimulated the formation of ELSs. In the case of both highly recalcitrant DH lines (DH19, DH119), properly developed ELSs were observed only in the presence of GSH. However, the number of produced ELSs was very low (Fig. 1) and regeneration of plants was not observed (Table 1).

**Table 1:** Plant regeneration in isolated microspore cultures of DH lines of winter triticale as the effect of various tiller pre-treatments (experiment 1*)*

DH line	Regeneration per spike	LT	LT + 3–8d GSH	LT + GSH
DH18	Total	3.8 ± 1.0^bcd^	16.8 ± 5.9^a^	16.7^a^
	Green plant	2.0 ± 0.5^b^	0.5 ± 0.3^b^	2.0^b^
DH19	Total	–	0.0	–
	Green plant	–	0.0	–
DH28	Total	11.8 ± 8.2^abc^	13.8 ± 1.2^ab^	12.6 ± 1.8^abc^
	Green plant	9.8 ± 6.2^a^	9.6 ± 1.1^a^	12.3 ± 4.2^a^
DH44	Total	0.6 ± 0.4^d^	9.3 ± 2.0^abcd^	3.5 ± 1.5^cd^
	Green plant	0.4 ± 0.4^b^	1.7 ± 1.0^b^	1.0 ± 1.0^b^
DH47	Total	4.4 ± 0.9^bcd^	1.0 ± 0.3^d^	10.0 ± 3.1^abcd^
	Green plant	0.9 ± 0.6^b^	0.0	3.0 ± 0.3^b^
DH101	Total	1.5 ± 0.6^d^	4.3 ± 2.0^bcd^	–
	Green plant	0.5 ± 0.3^b^	1.0 ± 0.6^b^	–
DH119	Total	–	–	0.0
	Green plant	–	–	0.0

Source of variation	Mean squares/*p*	
	Total regeneration	Green plant regeneration
DH line	176.128/***	102.652/***
Treatment	63.148/*	1.936/ns
DH line × treatment	33.911/*	1.722/ns

The mean from at least three biological replications (isolations) ± SE. Data marked with the same letter do not differ according to the Duncan test (*p* ≤ 0.05). The sources of variance for regeneration potential were as follows: seven DH lines, three treatments, and interaction between DH line and treatment. *, ***Significant at *p* ≤ 0.05, 0.001, respectively;  *ns* not significant

*LT* low temperature pre-treatment of tillers (21 days at 4 °C); *LT + 3–8d GSH* low temperature pre-treatment of tillers (21 days at 4 °C) combined with short GSH pre-treatment (0.3 mM GSH applied 3–8 days before microspore isolation); *LT + GSH* low temperature pre-treatment of tillers (21 days at 4 °C) combined with long GSH pre-treatment (0.3 mM GSH applied 21 days before microspore isolation)

‘–’ embryo-like structure was not developed

Short and/or long GSH pre-treatments increased the total number of regenerants produced per spike in a genotype-dependent manner, whereas green plant regeneration was not significantly changed (Table 1).

### Experiment 2

#### The effect of pre-treatment of tillers on the yield of microspore isolation, cell viability and its embryogenic potential

**Fig. 2:**
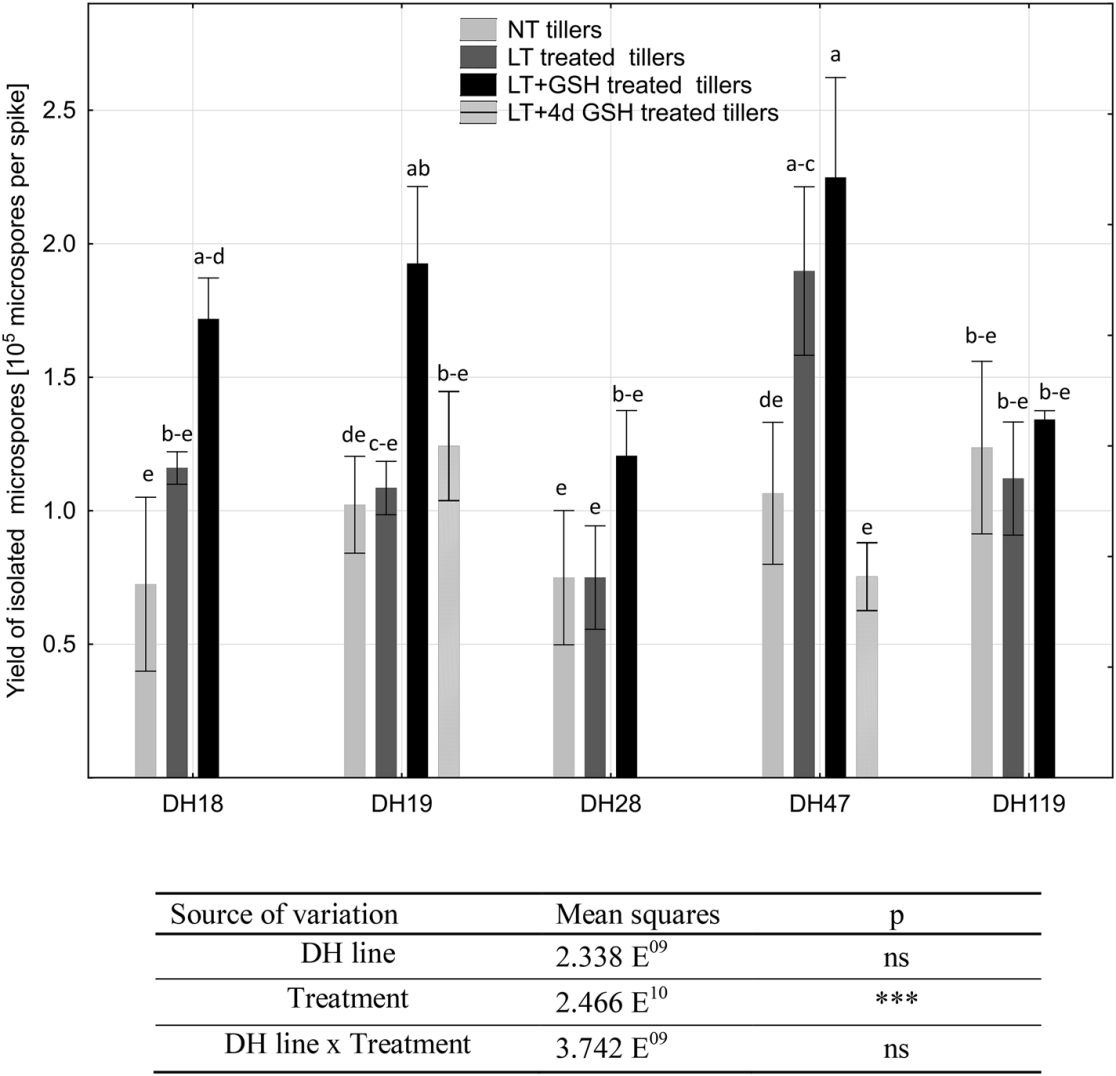
Isolated microspore yield (number of microspores isolated per spike of a donor plant) as the effect of various pre-treatments of tillers of DH lines of winter triticale. The mean from at least three biological replications (isolations) ± SE. Data marked with the same letter do not differ according to the Duncan test (*p* ≤ 0.05). The sources of variance for microspore yield were as follows: five DH lines, three treatments, and interaction between DH line and treatment. ***Significant at *p* ≤ 0.001; ns, not significant. *LT* low temperature pre-treatment of tillers (21 days at 4 °C); *LT + GSH* low temperature pre-treatment of tillers (21 days at 4 °C) combined with long GSH pre-treatment (0.3 mM GSH applied 21 days before microspore isolation). *LT + 4dGSH* low temperature pre-treatment of tillers (21 days at 4 °C) combined with short GSH pre-treatment (0.3 mM GSH applied 4 days before microspore isolation)

In the next experiment, a positive effect of LT + GSH pre-treatment on the isolated microspore yield was observed. Although in the majority of the studied DH lines this effect was observed only as a tendency (Fig. 2), the mean yield of isolated microspores increased significantly from 1.16 × 10^5^ to 1.62 × 10^5^ microspores per spike in comparison with the LT pre-treated tillers. Isolation from NT and LT + 4dGSH pre-treated tillers gave similar microspore yields to the control (ca. 0.97 × 10^5^ microspores per spike).

**Fig. 3:**
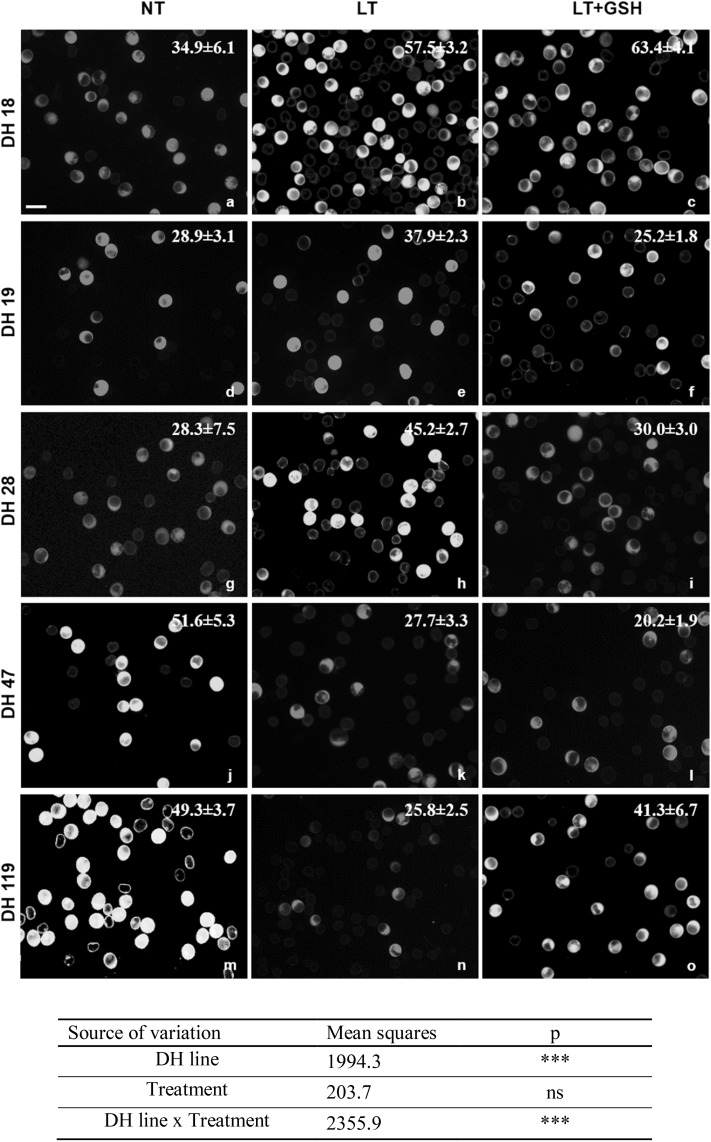
The effect of low temperature (LT) and glutathione (GSH) treatments on the viability of isolated microspores of DH lines of triticale (× *Triticosecale* Wittm.) with embryogenic potential. Viability of microspores isolated from tillers: non-treated (NT) (**a, d, g, j, m)**, treated with low temperature (LT) at 4 °C for 21 days (**b, e, h, k, n**), and after combined treatment with LT and reduced glutathione (LT + GSH) at 4 °C for 21 days (**c, f, i, l, o). a**–**o** Intensive green (FITC) caused by FDA staining demonstrates vital microspores. *Bar* 50 µm. The sources of variance for microspore viability were as follows: five DH lines, three treatments, and interaction between DH line and treatment. ***Significant at *p* ≤ 0.001; *ns* not significant

The number of fully viable microspores, measured by FDA staining just after the isolation procedure, ranged from 20.2 to 63.4% and ANOVA results showed that the values of these variables probably depended on the genotype of donor plants and on the interaction between the genotype and the method of the pre-treatment of tillers (Fig. 3). For two DH lines of triticale (DH47 and DH119), the highest cell viability was observed in microspore suspensions isolated from freshly cut, NT tillers. LT pre-treatment had a negative effect on microspore viability and almost halved the number of viable microspores (from 50.5 to 26.8%). In contrast, a positive effect of LT pre-treatment could be seen in responsive genotypes—DH18 and DH28—increasing the mean number of viable cells from ca. 32–51%. For two DH lines LT + GSH pre-treatment of tillers also significantly influenced microspore viability. However, both a positive (DH119) and a negative (DH28) effect was observed. Short GSH pre-treatment of tillers (LT + 4dGSH) applied to DH19 and DH47 had no effect on microspores viability.

**Fig. 4:**
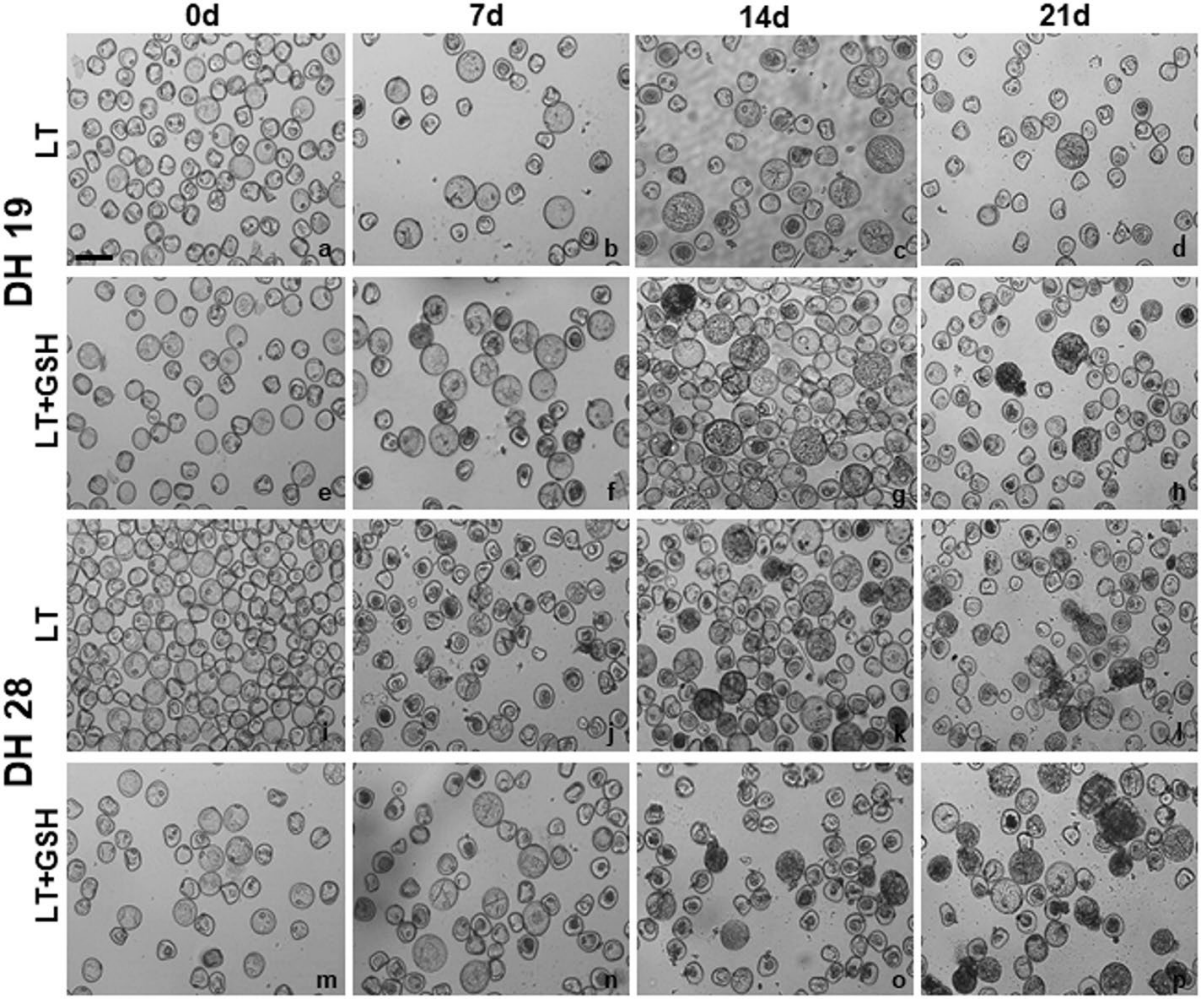
The effect of low temperature (LT) and glutathione (LT + GSH) treatments on embryo-like structures (ELSs) production in DH lines of triticale (× *Triticosecale* Wittm.) with different embryogenic potential. **a–h** Isolated microspores of the recalcitrant DH19 line after LT (**a–d**) and glutathione (LT + GSH; **e–h**) treatments. **i–p** Isolated microspores of the responsive DH28 line after LT (**i–l**) and glutathione (LT + GSH; **m–p**) treatments. **a, e, i, m** Microspore suspension on the isolation day (0d). **b, f, j, n** Multicellular structures after 7 days (7d) of in vitro culture. **c, g, k, o** Multicellular structures and ELSs after 14 days (14d) of in vitro culture. **d, h, l, p** ELSs after 21 days (21d) of in vitro culture. Bar 50 µm

**Fig. 5:**
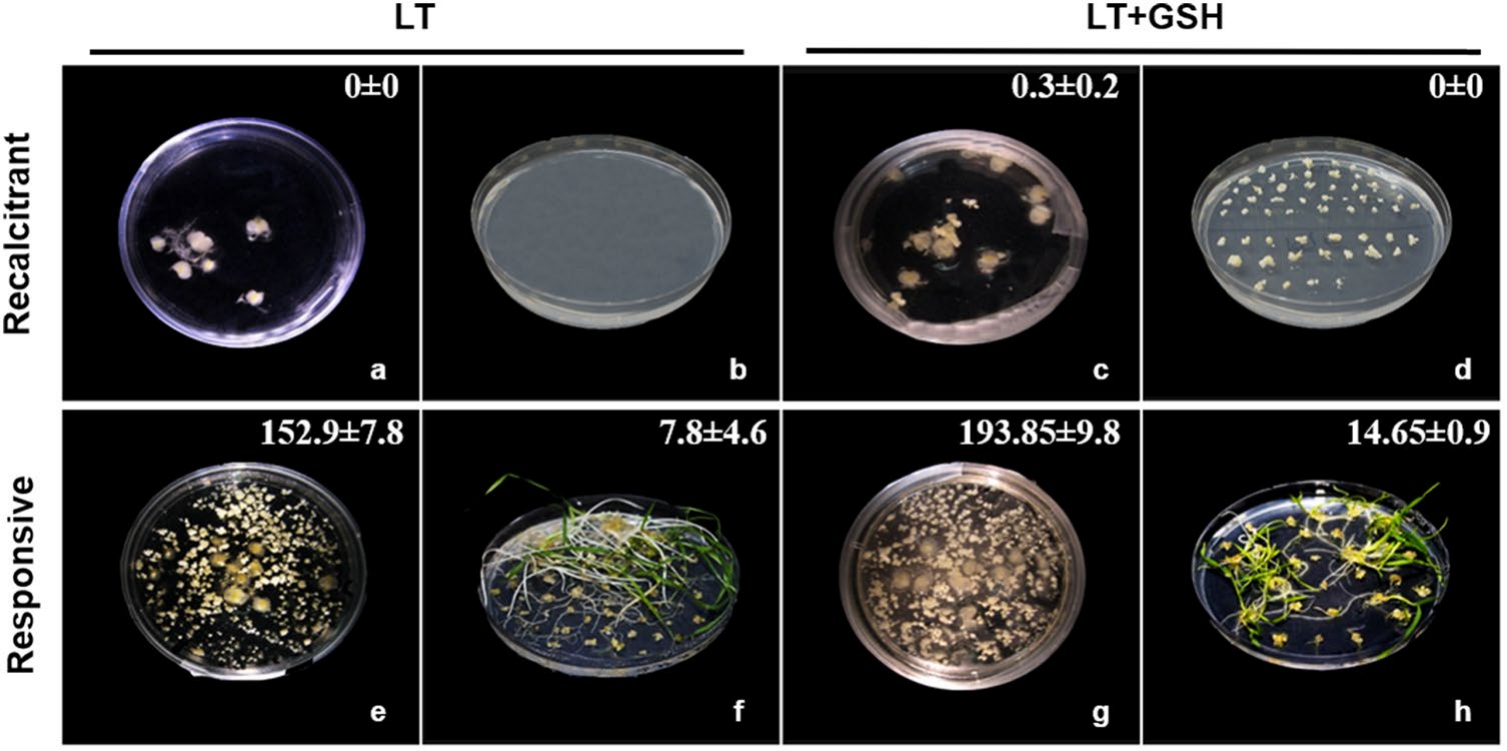
The effectiveness of ELS formation and total plant regeneration as the effect of stress pre-treatment applied to tillers of responsive and recalcitrant DH lines of triticale (**a–h**). Induction phase: embryo-like structures after 6 weeks of in vitro culture (**a, c, e, g**). Regeneration phase: green and albino plantlets derived from ELS on solid medium (**b, d, f, h**). Numeric data indicate the mean number of ELS (**a, c, e, g**) and the mean total number of regenerants (**b, d, f, h**) per spike of the donor plant ± SE. LT—low temperature pre-treatment of tillers (21 days at 4 °C); LT + GSH—low temperature pre-treatment of tillers (21 days at 4 °C) combined with long GSH pre-treatment (0.3 mM GSH applied 21 days before microspore isolation)

The effectiveness of ME induction expressed as the frequency of embryogenic microspores (EM), estimated on the isolation day, was significantly influenced by the genotype of donor plants (Table 2; Fig. 4). Based on this parameter, two DH lines (DH18, DH28) were considered as highly embryogenic with an average of 19% of microspores inducing embryogenic development under LT pre-treatment of tillers. Two others (DH19, DH119) confirmed their recalcitrance with only ca. 7% of EM observed after standard stress pre-treatment, whereas DH47 can be considered as moderate. The effect of LT + GSH pre-treatment was not significant, whereas LT + 4dGSH pre-treatment significantly increased the frequency of embryogenesis induction in DH19 (Table 2). Even though GSH had no effect on the number of EMs, microscopic observations showed that the development of ELS was faster. Starting from the 14th day LT + GSH-treated microspores were more advanced in development, forming clearly visible multicelluar structures and ELSs (Fig. 4). Efficient ELS formation and plant regeneration were achieved only for responsive DH lines of triticale. The effectiveness of total plant production depended significantly on pre-treatment of tillers (Fig. 5), whereas a similar number of ca. 6.5 green plants per spike of the donor plant was received.

**Table 2:** The frequency of embryogenic microspores^1^ in microspore suspension of DH lines of triticale on the day of isolation as the effect of various tiller pre-treatments (experiment 2)

DH line	LT	LT + GSH	LT + 4dGSH
DH119	7.4 ± 1.3^bcd^	3.6 ± 1.0^d^	–
DH19	7.5 ± 2.5^bcd^	2.2 ± 2.2^d^	20.8 ± 4.6^a^
DH47	16.6 ± 7.2^abc^	6.6 ± 2.7^cd^	13.9 ± 2.4^abc^
DH28	18.3 ± 2.2^ab^	17.3 ± 3.5^abc^	–
DH18	21.1 ± 9.9^a^	13.1 ± 1.7^abcd^	–

Source of variation	Mean squares/*p*
DH line	532.186/***
Treatment	515.555/*
DH line × treatment	42.882/ns

The mean from at least three biological replications (isolations) ± SE. Data marked with the same letter do not differ according to the Duncan test (*p* ≤ 0.05). The sources of variance for embryogenic potential were as follows: five DH lines, three treatments, and interaction between DH line and treatment

*LT* low temperature pre-treatment of tillers (21 days at 4 °C); *LT + 3–8d GSH* low temperature pre-treatment of tillers (21 days at 4 °C) combined with short GSH pre-treatment (0.3 mM GSH applied 3–8 days before microspore isolation); *LT + GSH* low temperature pre-treatment of tillers (21 days at 4 °C) combined with long GSH pre-treatment (0.3 mM GSH applied 21 days before microspore isolation)

*, ***Significant at *p* ≤ 0.05, 0.001, respectively; *ns* not significant

^1^The percentage of star-like structures (SLS) and microspores undergoing the first symmetrical nucleus division

‘–’ 4-day GSH pre-treatment was not applied

#### The effect of pre-treatment of tillers on the endogenous level of reduced glutathione, glutathione redox status and total non-enzymatic antioxidant activity

**Fig. 6:**
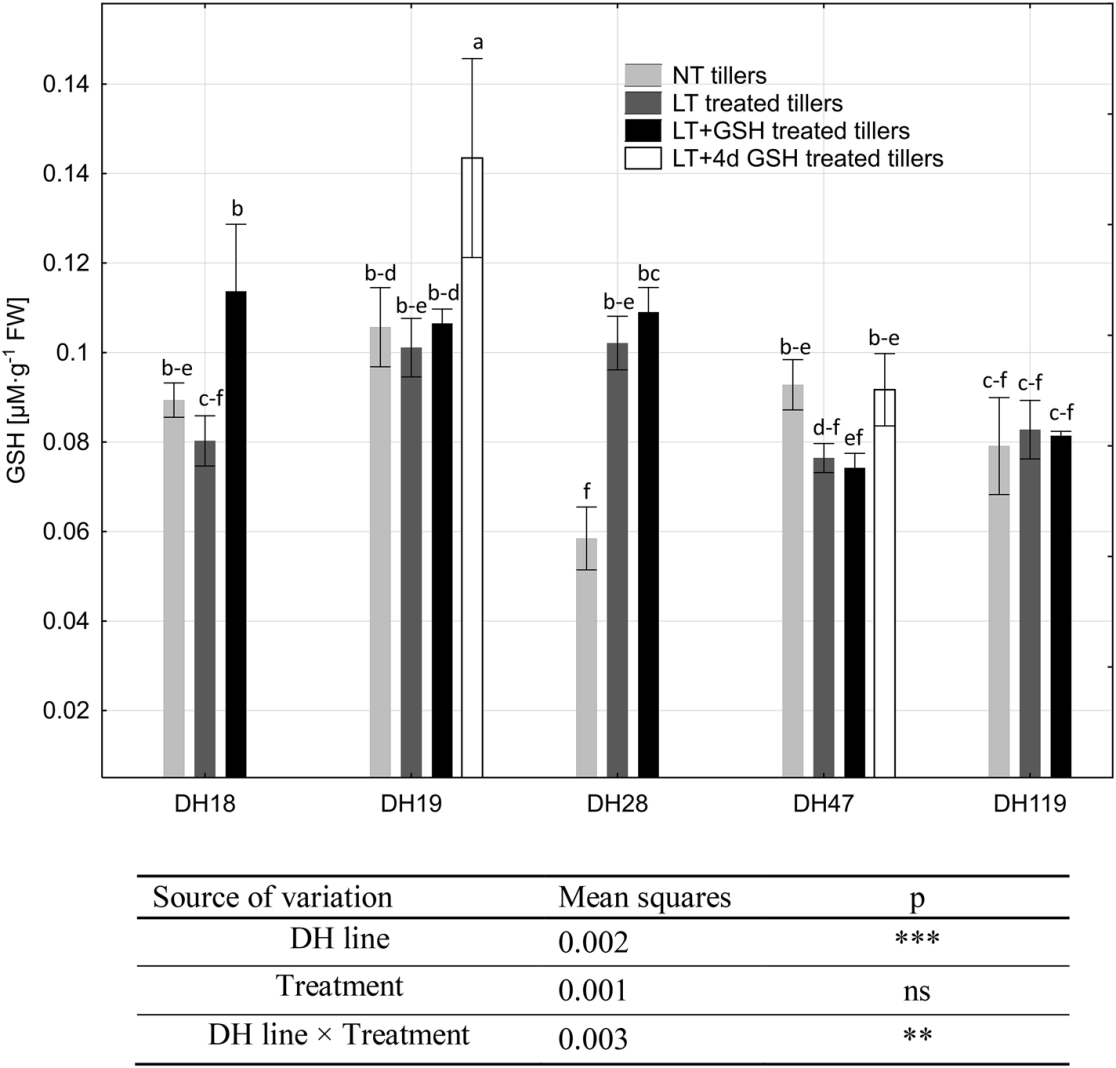
Endogenous level of reduced glutathione (GSH) in triticale microspores isolated after various pre-treatments of tillers. The mean from at least three biological replications (isolations) ± SE. Data marked with the same letter do not differ according to the Duncan test (*p* ≤ 0.05). The sources of variance for endogenous level of reduced glutathione (GSH) were as follows: five DH lines, three treatments, and interaction between DH line and treatment. **, ***Significant at *p* ≤ 0.01, 0.001, respectively; *ns* not significant. *LT* low temperature pre-treatment of tillers (21 days at 4 °C); *LT + GSH* low temperature pre-treatment of tillers (21 days at 4 °C) combined with long GSH pre-treatment (0.3 mM GSH applied 21 days before microspore isolation). *LT + 4dGSH* low temperature pre-treatment of tillers (21 days at 4 °C) combined with short GSH pre-treatment (0.3 mM GSH applied 4 days before microspore isolation)

The endogenous level of GSH in microspores ranged from 0.058 to 0.143 µM·g^−1^ FW and ANOVA results showed that the values of these variables probably depended on the genotype and the interaction between the genotype and tiller pre-treatment (Fig. 6). Analysing each DH line separately, a significant effect of tiller pre-treatment was observed only in a few cases. LT pre-treatment increased endogenous GSH level in microspores of DH28, whereas LT + GSH pre-treatment resulted in GSH accumulation in both responsive DH lines (DH18 and DH28). Significantly higher GSH content was also detected in microspores of DH19 after LT + 4dGSH pre-treatment. Analysing DH lines grouped according to their embryogenic potential, it could be observed that in the case of highly recalcitrant genotypes (DH19 and DH119), LT and LT + GSH pre-treatments of tillers had no effect on endogenous GSH content, whereas in microspores of responsive DH lines (DH18 and DH28), endogenous GSH content increased significantly from 0.077 to 0.095 µM·g^−1^ FW in response to LT pre-treatment, and to 0.110 µM·g^−1^ FW under LT + GSH pre-treatment.

**Fig. 7:**
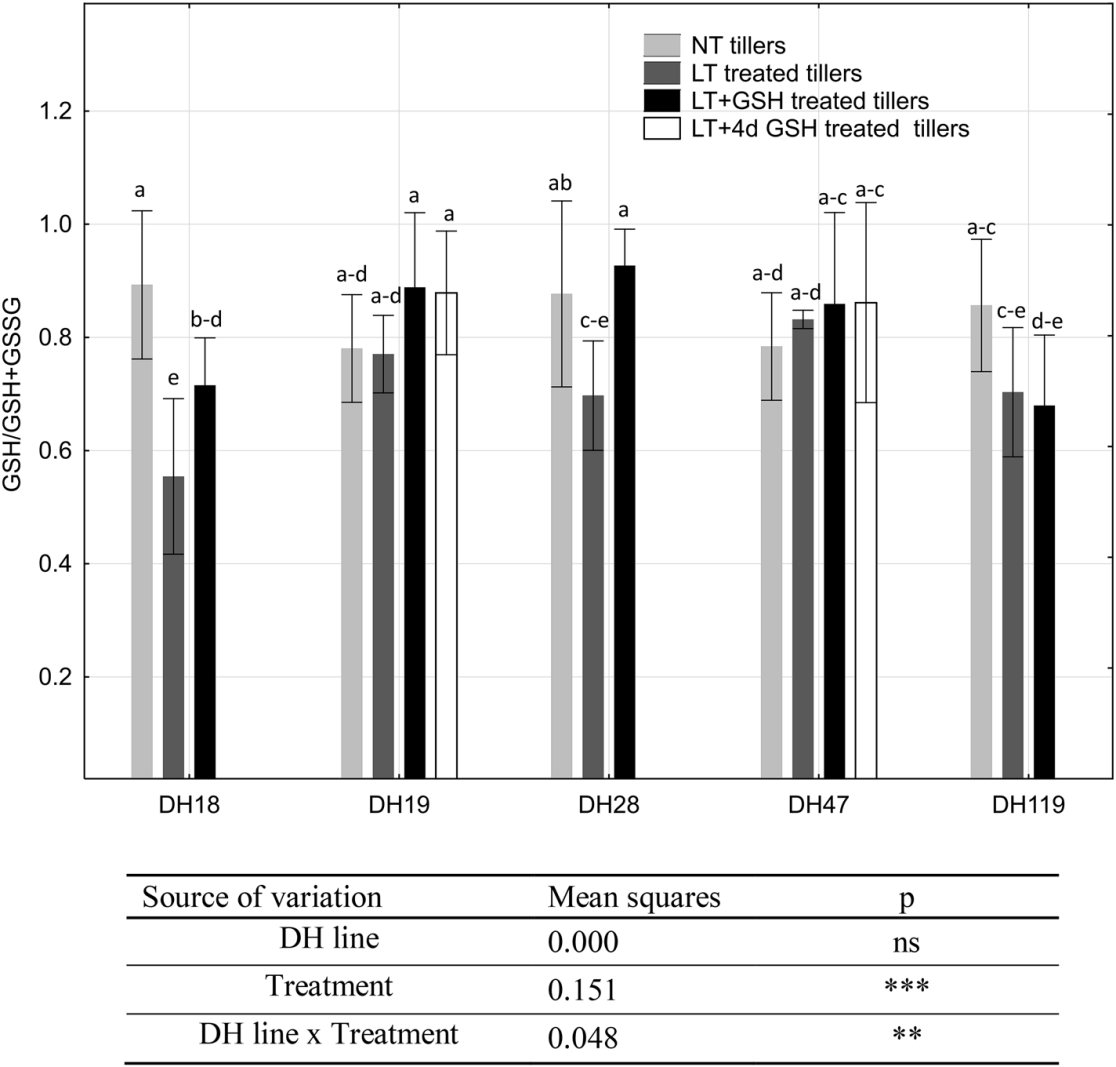
Glutathione redox status in triticale microspores isolated after various pre-treatments of tillers. The mean from at least three biological replications (isolations) ± SE. Data marked with the same letter do not differ according to the Duncan test (*p* ≤ 0.05). The sources of variance for glutathione redox status were as follows: five DH lines, three treatments, and interaction between DH line and treatment. **, ***Significant at *p* ≤ 0.01, 0.001, respectively; *ns* not significant. *LT* low temperature pre-treatment of tillers (21 days at 4 °C); *LT + GSH* low temperature pre-treatment of tillers (21 days at 4 °C) combined with long GSH pre-treatment (0.3 mM GSH applied 21 days before microspore isolation). *LT + 4dGSH* low temperature pre-treatment of tillers (21 days at 4 °C) combined with short GSH pre-treatment (0.3 mM GSH applied 4 days before microspore isolation)

The most pronounced differences in the microspore redox status, estimated as GSH/(GSH + GSSG) ratio, were observed in the case of two highly responsive DH lines of triticale—DH18 and DH28 (Fig. 7). LT pre-treatment of tillers induced a significant decrease in GSH/GSH + GSSG parameter, whereas application of exogenous GSH erased or diminished this effect. One more significant difference was observed between microspores of DH119 isolated from NT tillers and LT + 4dGSH treated tillers, with a greater pool of the reduced form of glutathione detected in microspores isolated from freshly cut (NT) tillers.

**Fig. 8:**
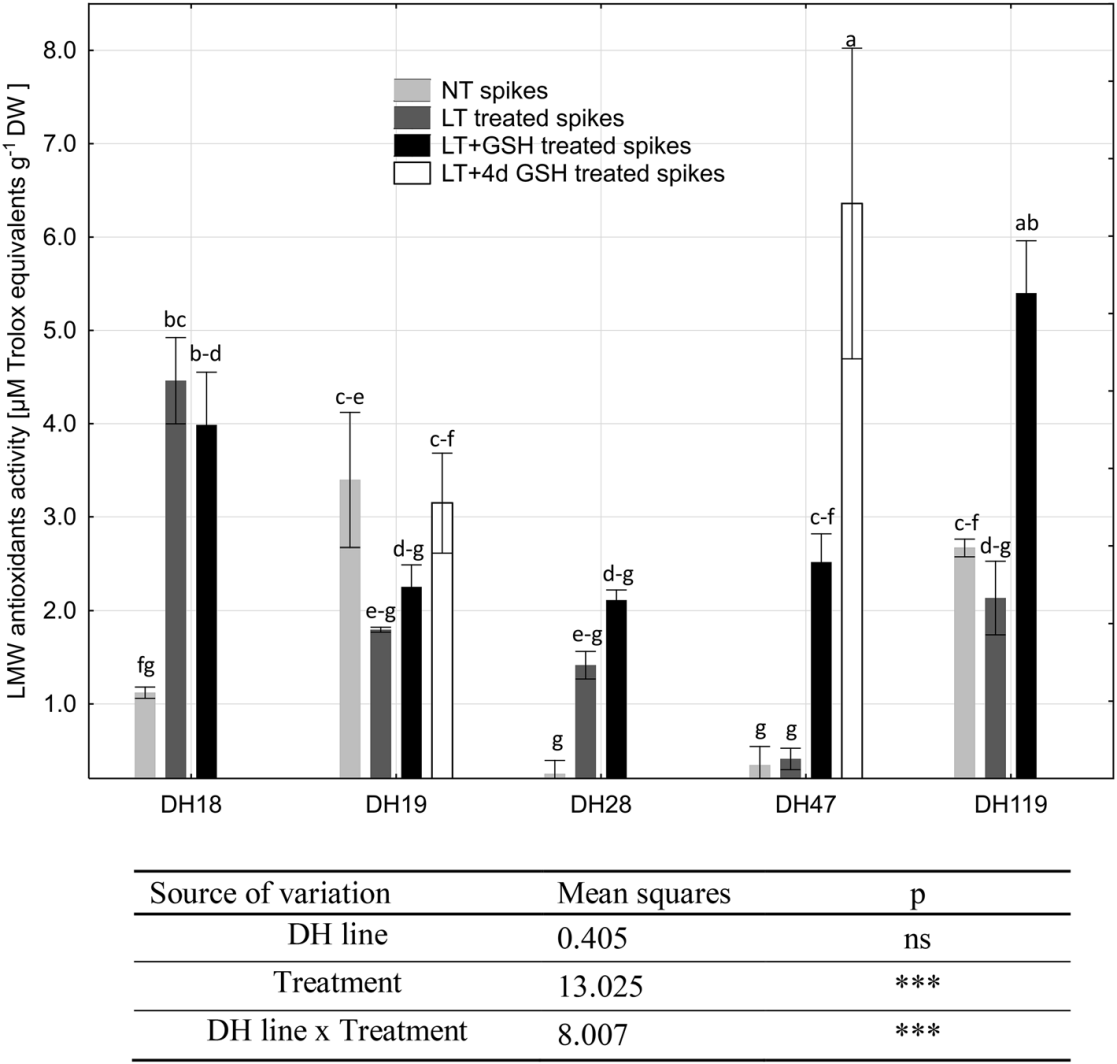
Activity of low-molecular weight (LMW) antioxidants in triticale microspores isolated after various pre-treatment of tillers. The mean from at least three biological replications (isolations) ± SE. Data marked with the same letter do not differ according to the Duncan test (*p* ≤ 0.05). The sources of variance for activity of LMW antioxidants were as follows: five DH lines, three treatments, and interaction between DH line and treatment. ***Significant at *p* ≤ 0.001, respectively; *ns*, not significant. *LT* low temperature pre-treatment of tillers (21 days at 4 °C); *LT + GSH* low temperature pre-treatment of tillers (21 days at 4 °C) combined with long GSH pre-treatment (0.3 mM GSH applied 21 days before microspore isolation). *LT + 4dGSH* low temperature pre-treatment of tillers (21 days at 4 °C) combined with short GSH pre-treatment (0.3 mM GSH applied 4 days before microspore isolation)

Total activity of LMW antioxidants varied between 0.25 and 6.36 µM Trolox eqv. g^−1^ DW and ANOVA results showed that the values of these variables probably depended on tiller pre-treatment (Fig. 8). Strong interaction between the genotype of donor plants, its embryogenic potential and LMW activity was also revealed. The lowest LMW antioxidant activity was detected in microspores of two responsive DH lines (DH18 and DH28) and DH47 isolated from freshly cut (NT) tillers (0.25–1.12 µM Trolox eqv. g^−1^ DW). LT pre-treatment of tillers significantly influenced antioxidative activity but only in microspores of responsive DH lines, increasing its mean value from 0.69 to 3.32 µM Trolox eqv. g^−1^ DW. The combined LT + GSH pre-treatment induced the same effect, increasing LMW antioxidant activity to 2.82 µM Trolox eqv. g^−1^ DW.

In contrast, high LMW antioxidant activity was detected in microspores of two highly recalcitrant DH lines—DH19 and DH119—isolated from NT tillers (3.09 µM Trolox eqv. g^−1^ DW). LT pre-treatment did not induce any significant effect either in microspores of highly recalcitrant DH lines or in DH47. However, LT combined with GSH pre-treatment significantly stimulated antioxidative defence in DH119 and DH47, especially in microspores of DH47 isolated after LT + 4dGSH pre-treatment (6.36 µM Trolox eqv. g^−1^ DW).

#### Analysis of Spearman’s rank-order correlation coefficients (*R*)

Analysis of Spearman’s Correlation coefficients (*R*) was used separately for DH lines of triticale significantly different in respect of their embryogenic potential. Significant correlation (*R* = − 0.83) was revealed only between the frequency of embryogenic microspores and the activity of LMW antioxidants in recalcitrant DH lines of triticale.

## Discussion

The studied DH lines of winter triticale were selected from the mapping population ‘Saka 3006’ × ‘Modus’ derived using the method of distant crosses with maize (Wędzony 2003; Tyrka et al. 2011). The population composed of 90 DH was carefully examined in respect of its embryogenic potential by anther culture method and used as the model in our earlier studies focused on physiological, metabolomic and molecular aspects of ME (Żur et al. 2012, 2014a, b, 2015; Krzewska et al. 2012, 2015, 2017). Seven DH lines selected for the first experiment were significantly different in respect of their embryogenic potential (Żur et al. 2014a, b, 2015). The efficiency of ME in isolated microspore cultures after standard pre-treatment of triticale tillers (Pauk et al. 2000) confirmed the earlier characterization of DH19 and DH119 as highly recalcitrant, and DH28 as highly embryogenic genotypes. Four other DH lines (DH18, DH44, DH101 and DH47) turned out to be much less responsive in isolated microspore cultures (4.3–29.2 ELS per spike) in comparison with anther culture method (60.4-130.4 ELS per 100 anthers, assuming that one spike gives approximately 100–120 anthers).

The results received in the first experiment confirmed the importance of LT pre-treatment for sustaining relatively high microspore viability during the isolation procedure and after the transfer to in vitro culture. They also indicated that exogenous GSH applied during LT pre-treatment of tillers distinctly increased the effectiveness of ME. Of particular significance was the moment of GSH application and the duration of the treatment. While short-term GSH pre-treatment applied 3–8 days before microspore isolation significantly increased the efficiency of ELS production in the majority of the studied DH lines of triticale, the effect of long GSH pre-treatment was not so pronounced but showed a tendency to promote the regeneration of plants. These results seem to confirm positive effects of exogenous antioxidants on the process of ME reported earlier by Cistué et al. (2009), Asif et al. (2013), Hoseini et al. (2014) and Zeng et al. (2017). However, the stimulation of green plant regeneration observed in the above-mentioned experiments was not confirmed. Moreover, in contrast to Asif et al. (2013), such medium supplementation did not induce any significant effect on final ME effectiveness in the case of the DH lines of triticale used in this study (data not shown). It could be speculated that conversion of proplastids into chloroplasts is induced by some signalling molecules (e.g., ROS) and that GSH treatment directly or through a change in cell redox potential disturbs the pathway of signal transduction and/or further ELS development. However, a more prosaic explanation could also be given, as a higher number of developing ELSs compete more severely for nutrients and oxygen, and release higher amounts of toxic by products of cell metabolism. Even regular refreshment of the medium cannot totally obviate the problem, which could result in smaller size and disturbances in development observed after 6 weeks of in vitro culture in a part of ELS population.

As the most abundant low-molecular weight antioxidant accumulated at high concentrations in most cells, glutathione is a very important element of the antioxidative system, protecting cells from the effects of excessive ROS generation (Noctor et al. 2012). As the main redox buffer in a cell, it has a significant influence on intracellular redox homeostasis, changes of which affect cell signalling, gene transcription, translation, cell proliferation, and cell death. A high level of GSH, which ensures intracellular reducing environment, prevents protein thiol oxidation and ensures the correct protein folding and functioning. Under stress conditions, such as cold, the ratio of GSH in total intracellular glutathione pool decreases because GSH is used for ROS detoxification in the ascorbate–glutathione cycle (Foyer et al. 1997). Accumulation of GSH at low non-freezing temperatures was reported in several plant species, suggesting its involvement in cold acclimation (review in Kocsy et al. 2001). Studies using *A. thaliana cat2* mutant characterized by low GSH/GSSG ratio have shown that expression of genes regulating salicylic acid and jasmonic acid signal transduction depend on GSH availability (Gao et al. 2013). However, glutathione’s function is not restricted to antioxidative activity and its role played in plant development cannot be replaced by any other antioxidants. The crucial role of glutathione in embryo and meristem development was confirmed by the analysis of the phenotypes of glutathione-deficient *Arabidopsis* mutants (Cairns et al. 2006; Noctor et al. 2012). Knocking out expression of gene-encoding enzymes involved in glutathione synthesis causes lethality at the embryo or seedling stage. Recently published results (Schnaubelt et al. 2015) have indicated several GSH-responsive genes coding transcription factors (SPATULA, MYB5, MYB75) and proteins involved in the regulation of cell divisions, redox potential (e.g., thioredoxins, glutaredoxin) and auxin biosynthesis, transport and transcriptional response (HECATE). The effects of exogenous glutathione and endogenous glutathione redox status were examined in various—including in vitro*—*systems, confirming its important role in somatic and microspore-derived embryo development (Stasolla and Yeung 2003; Stasolla et al. 2004; Belmonte et al. 2006; Cistué et al. 2009). It was revealed that initial stages of embryogenesis required reduced environment (high GSH/GSH + GSSG), which promotes cell proliferation, possibly by enhanced synthesis of nucleotides and mitotic activity (De Gara et al. 2003; Stasolla and Yeung 2003; Belmonte et al. 2005; Stasolla 2010). The next stages of embryo growth and differentiation, connected with the accumulation of storage products, were promoted by decreased redox status of the glutathione pool. Application of GSH during the second half of somatic embryo development results in precocious germination (Belmonte et al. 2005), whereas GSSG supplemented to the maturation medium resulted in improved somatic embryogenesis (Belmonte and Yeung 2004; Belmonte et al. 2005). Similarly, application of buthionine sulfoximine (BSO), an inhibitor of glutathione de novo synthesis, to microspore-derived embryos of rapeseed enhanced embryo conversion frequency, which was ascribed to an increased level of endogenous ABA resulting in better structural organization of the shoot apical meristem (Belmonte et al. 2006).

**Fig. 9:**
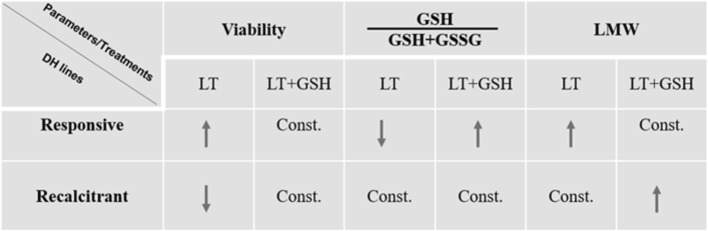
Summary. Changes in microspores viability, ratio between reduced and oxidized forms of glutathione (GSH/GSH + GSSG) and low-molecular weight antioxidants (LMW) activity induced by ME-inducing pre-treatment of tillers (LT, LT + GSH) in responsive/recalcitrant DH lines of triticale (× *Triticosecale* Wittm.). Arrows indicate the predominant direction of changes: increase (↑), decrease (↓) or constant (Const.). *LT* low temperature pre-treatment of tillers (21 days at 4 °C); *LT + GSH* low temperature pre-treatment of tillers (21 days at 4 °C) combined with long GSH pre-treatment (0.3 mM GSH applied 21 days before microspore isolation)

**Fig. 10:**
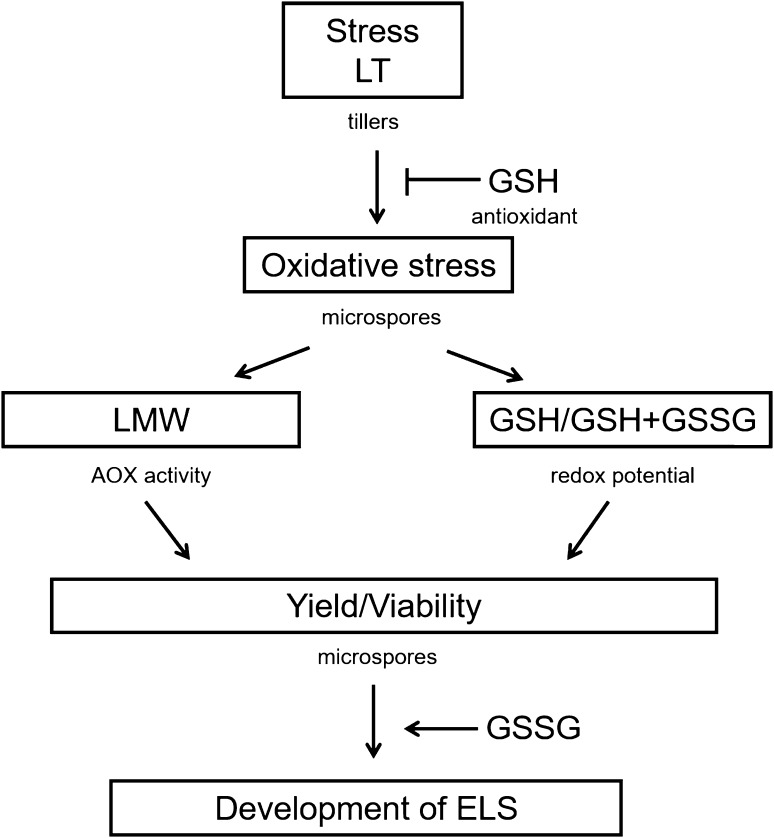
Flow chart. Possible roles of glutathione in microspore embryogenesis (ME) in triticale (× *Triticosecale* Wittm.). Low temperature (LT) pre-treatment of tillers induces microspore reprogramming and results in significant changes (1) in the activity of low-molecular weight antioxidants (LMW) and (2) in the ratio between reduced glutathione (GSH) and the pool of both reduced and oxidized forms (GSH/GSH + GSSG). Exogenously applied GSH (antioxidant) protects cells from oxidative damages, which influences microspore yield, viability and the effectiveness of microspore embryogenesis (ME). Increased endogenous level of GSSG promotes further embryo-like structure (ELS) development in isolated microspore cultures

The presented, more detailed examination of the effects of GSH pre-treatment with the use of five DH lines of triticale gave more information about the mechanism of ME induction and confirmed the dual role of glutathione in ME stimulation (Figs. 9, 10).

The results of microscopic observations and biochemical analyses confirmed the responsive/recalcitrant character of the four genotypes (DH28, DH18/DH19, DH119) with significantly different pattern of response specific for DH47. The received results also confirmed that the main difference between recalcitrant and responsive DH lines is the level of tolerance to stresses connected with ME-inducing pre-treatment and the procedure of microspore isolation. The observed lower activity of LMW antioxidants detected in microspores isolated from control NT tillers of responsive DH lines suggests a lower level of oxidative stress probably due to more efficient enzymatic antioxidative defence (Żur et al. 2014a). The effect of LT pre-treatment, standardly used in triticale as ME-inducing factor, was significantly different. In responsive DH lines, LT significantly increased the viability of microspores and induced ME in approximately 20% of isolated cells, which was associated with significantly higher LMW antioxidant activity and a significant decrease in cell redox status (GSH/GSH + GSSG) in comparison with NT microspores. It suggests that responsive DH lines more efficiently utilise GSH in response to LT pre-treatment, probably in defence reactions protecting cells from the results of oxidative stress. Exogenous GSH applied at the start of LT pre-treatment of tillers induced a stable effect on the accumulation of endogenous GSH and its redox status, although it was not associated with increased antioxidative activity and had no or negative effect on microspore viability and the initial frequency of embryogenic microspores. At the same time, however, it promoted ELS formation, as could be seen on the basis of microscopic observations and the results of both experiments.

In the case of two highly recalcitrant DH lines, LT pre-treatment of tiller was also necessary for successful transfer of microspores to in vitro conditions and resulted in ME initiation in approximately 7.5% of isolated cells. Conversely, LT treatment had no effect on or decreased microspore viability. It was probably the result of a decrease in antioxidative enzyme activity known from an earlier study (Żur et al. 2014a). Moreover, enhanced activity of LMW antioxidants, revealed earlier in isolated anthers, was not detected in isolated microspores, which could explain, at least partially, lower effectiveness of ME in isolated microspore cultures in comparison with anther cultures. Probably that is also why the supplementation of exogenous GSH can increase the number of microspores able to survive LT pre-treatment and isolation procedure. However, the effect of GSH was the result of the interaction between the genotype and tiller pre-treatment. Depending on the moment of application, exogenous GSH could induce a stable increase in LMW antioxidant activity and sustain microspore viability without any effect on the effectiveness of ME induction or significantly increase the level of endogenous GSH, which was associated with an increased number of embryogenic microspores. However, even in the case when LT + 4dGSH treatment enhanced antioxidative activity and sustained cell viability, which resulted in a high frequency of ME initiation, it was still insufficient for a really efficient haploid/doubled haploid plant production. Not only was the number of produced ELS too small, but also their regeneration failed.

Generally, a positive effect of long GSH pre-treatment on the isolated microspore yield was observed. Interestingly, higher yield of isolated microspores was not synonymous with higher viability of FDA-positive microspores. This observation was confirmed by microscopic analysis, and suggests that a part of GSH-treated microspores that survived the isolation procedure were not at the proper stage of development and started to die almost immediately after transfer to in vitro culture conditions.

To summarize, the data presented here are in agreement with the results received in our earlier study with the use of the anther culture method (Żur et al. 2014a), which showed that ROS generation associated with efficient antioxidative defence is the first, although not the only, prerequisite for effective ME initiation. An increasing number of recently published data indicate that ROS accumulation starts signal transduction leading to microspore reprogramming and embryogenic development. LT pre-treatment of microspores equipped with efficient, stress-resistant antioxidative system protecting the cells from damage connected with escalated oxidative reactions, efficiently induces the process of ME. As additional antioxidative protection seems to be unnecessary, the observed enhanced effectiveness of ME is probably the effect of the specific role of glutathione in the stimulation of ELS development. For microspores of recalcitrant genotypes, in which the activity of the antioxidative system decreases significantly during LT pre-treatment, any factor increasing stress adaptation ability seems to be of special importance. In this case, the major role of exogenous GSH is the support of inefficient inner system in cell protection against oxidative stress. However, excessive ROS elimination probably suppresses the signal necessary for microspore reprogramming. Moreover, reduced environment can either negatively influence further ELS development or impair their ability to regenerate into plants. It could also be supposed that there are still other defective elements in the very complex network that coordinates and regulates the process of ME. The proposed hypothesis explains why the effects of exogenously applied antioxidants can vary significantly depending on the plant genotype, plant condition and its interaction with environmental factors, though its verification requires further, more detailed investigations.

### Author contributions

IŻ conceived and designed the research, conducted experiments and wrote the manuscript. ED and MK conducted experiments. FJ measured antioxidative activity. KZ measured glutathione content. JF contributed to glutathione analysis. All authors read and approved the manuscript.

## Electronic supplementary material

Below is the link to the electronic supplementary material.

**Online resource 1** Scheme of the experiment. Microspores isolated from non-treated (NT) or stress pre-treated (LT, LT+GSH, LT+4dGSH) tillers were analysed in respect of: (1) isolated microspore yield, (2) microspore viability, (3) the endogenous level of reduced glutathione (GSH) together with (4) the ratio between reduced and the pool of both reduced and oxidized forms of glutathione (GSH/GSH+GSSG) (5) antioxidative activity of low-molecular weight antioxidants (LMW AOX) and (6) the frequency of embryogenic microspores. The majority of microspores isolated from NT tillers were at late uni-nucleate stage (LU) of development. Microspores isolated from stress pre-treated tillers (LT+GSH: DH18, DH19, DH28, DH47, DH119; LT+4dGSH: DH19, DH47) exhibited star-like (SLS) morphology (typical hallmark of embryogenesis initiation) with genotype-dependent frequency (TIF 7737 KB)
